# Saline–alkaline stress in growing maize seedlings is alleviated by* Trichoderma asperellum* through regulation of the soil environment

**DOI:** 10.1038/s41598-021-90675-9

**Published:** 2021-05-27

**Authors:** Jian Fu, Yao Xiao, Yu-feng Wang, Zhi-hua Liu, Kejun Yang

**Affiliations:** 1 Key Laboratory of Saline-Alkali Vegetation Ecology Restoration, Ministry of Education (Northeast Forestry University), Harbin, 150040 People’s Republic of China; 2 College of Agronomy, Heilongjiang Bayi Agricultural University/Key Laboratory of Modern Agricultural Cultivation and Crop Germplasm Improvement of Heilongjiang Province, Daqing, 163319 Heilongjiang Province People’s Republic of China; 3 Postdoctoral Research Station for Crop Science of Heilongjiang, Bayi Agricultural University, Daqing, 163319 People’s Republic of China; 4 College of Horticulture and Landscape Architecture, Heilongjiang Bayi Agricultural University, Daqing, 163319 People’s Republic of China; 5 College of Forestry, Shenyang Agricultural University, Shenyang, 110866 People’s Republic of China

**Keywords:** Plant physiology, Agroecology

## Abstract

A significant proportion of the land area of Heilongjiang Province, China, is composed of saline–alkaline soil, which severely inhibits maize growth. Although *Trichoderma* treatment is widely regarded as a promising strategy for improving the soil environment and promoting plant growth, the mechanism through which *Trichoderma asperellum* enhances maize resistance to saline–alkaline stress is not clear. In this study, we explored the effect of *T. asperellum* application at different concentrations to soil saline–alkaline environment on the seedlings of two maize cultivars, assessing the biochemical parameters related to oxidation resistance. Increasing spore densities of *T. asperellum* suspension effectively regulated the soil ion balance in the rhizosphere of maize seedlings, reduced the soil pH by 2.15–5.76% and sodium adsorption ratios by 22.70–54.13%, increased soil nutrient content and enzyme activity, and improved the soil environment for seedling growth. Additionally, *T. asperellum* treatment increased the maize seedling content of osmo-regulating substances and rate of glutathione:oxidised glutathione (43.86–88.25%) and ascorbate:oxidised ascorbate (25.26–222.32%) by affecting the antioxidant enzyme activity in the roots, increasing reactive oxygen species scavenging, and maintaining the osmotic balance and metabolic homeostasis under saline–alkaline stress. *T. asperellum* also improved the saline–alkaline tolerance of maize seedlings by improving the root growth characteristics. Moreover, results showed that *Trichoderma* applied at high concentration had the greatest effect. In conclusion, improvement in the saline–alkaline tolerance of maize seedlings by *T. asperellum* under saline–alkaline soil conditions may be achieved through diverse effects that vary among maize cultivars.

## Introduction

As global human population is expected to increase to nearly 10 billion over the next 50 years, meeting the worldwide food demand, i.e., a fundamental social need, will require at least a 50% increase in global grain production^1^. Meanwhile, saline–alkaline soil covers over 954 million ha worldwide^2^ and is rapidly increasing every year. Further, the increase in soil salinization is caused by the current heavy use of fertilizers and causes great economic losses for agricultural productivity^3–5^. This affects the sustainable development of agricultural ecosystems and, ultimately, threatens food security.

In China, Heilongjiang Province is one of the most important maize production areas, with predominantly saline–alkaline soils (2.882 × 10^6^ ha) that seriously restrict the average grain yield^6^. Maize shows medium sensitivity to salinity and alkalinity, its tolerance to these conditions varies considerably among cultivars, and its yield can be reduced by 20–46% when grown on saline–alkaline soil^7^. Excess salt concentration degrades the soil, changes soil permeability and substrate potential, and decreases soil microbial activity^8^. Moreover, soil saline–alkaline stress can cause roots to suffer from ion poisoning and osmotic imbalance, destroy root cell structure, and significantly reduce root vigor, therefore inhibiting crop growth and resulting in a significant reduction in plant biomass, increased plant wilt, and death^9^.

Under saline–alkaline stress, substantial amounts of reactive oxygen species (ROS) are produced in plants, leading to gradual peroxidation of lipids and changes in antioxidant enzyme activities^7^. To remove and detoxify excess ROS, plants form both enzymatic and non-enzymatic antioxidant defenses. The ascorbate–glutathione (AsA–GSH) cycle has an important function in eliminating H_2_O_2_ and can be stimulated in plants by moderating stress conditions to scavenge ROS^10^. AsA and GSH are two low molecular weight antioxidants involved in reactions in the AsA–GSH cycle and important for preserving a wide range of metabolic processes^11^. Hence, maintaining high GSH/oxidized glutathione (GSSG) and AsA/oxidized ascorbic acid (DHA) ratios is crucial for enhancing the saline–alkaline stress tolerance of plants. Furthermore, harmful saline–alkaline soil ions cause a significant decrease in soil enzyme activity by directly inhibiting the number of microorganisms in the soil^12^. Crops growing in saline–alkaline soils suffer Na^+^ toxicity and high pH stress caused by excess Na_2_CO_3_ and NaHCO_3_, which are known to cause greater damage than NaCl^6^. To minimize these negative effects of saline–alkaline soils that have been contaminated owing to intensive farming practices, organic technology can be combined with microbes for synergistic plant-growth enhancement or soil bioremediation^13^.

*Trichoderma* is an important fungus that can spread rapidly in the soil, colonizing and surviving on the surface of plant root systems for prolonged periods, proliferating, and forming effective groups for biocontrol in crops and soils. *Trichoderma* effectively promotes root growth and the secretion of organic compounds that induce local or systemic resistance in plants^14^ and has a strong ability to mobilize and absorb soil nutrients. Thus, compared to other soil microbes, *Trichoderma* is more efficient and competitive in effectively improving soil structure, increasing the nutrient utilization efficiency by crops, and promoting crop growth^15,16^. For example, *Trichoderma* treatments improves the growth of *Arabidopsis thaliana* seedlings under salt stress by increasing root development and producing osmotic substances to eliminate Na^+^^17^. Similarly, *Trichoderma harzianum* significantly alleviates the salinity effects on tomato growth under saline irrigation and has a positive effect on the effective phosphorus concentration in the soil, which effectively reduces the need for phosphorus fertilizer^18^. Furthermore, Yasmeen and Siddiqui indicated the ameliorative effects of *T. harzianum* (Th-6) on maize and rice under a hydroponic saline environment, showing that *Trichoderma* promotes plant growth and increases environmental stress tolerance^19^. They also identified several interaction mechanisms that take place among the plants, soil, and *Trichoderma* under salt stress, such as *Trichoderma* increasing the activity of antioxidative defense systems in plants to resist salt stress and enhancing the relative antioxidant gene expression levels in stressed plants^20,21^.

Abiotic stress in *Trichoderma*-treated plants has been studied on vegetables, maize, wheat, and soybean, among others, with most studies artificially simulating the abiotic stress conditions of interest, such as drought or salinity. Owing to the complexity of natural saline–alkaline soil conditions, it is difficult to effectively simulate such conditions artificially. Thus, in this study, an experiment was performed in a natural saline–alkaline soil. To our knowledge, there are no reports on the effect of *Trichoderma* on soil characteristics and AsA–GSH cycle in maize under saline–alkaline soil stress conditions. This study evaluated the soil salt ion content, sodium adsorption ration (SAR), and pH value; soil enzyme and nutrients contents, antioxidant enzyme activities, non-enzymatic antioxidant (i.e., AsA and GSH) contents and redox ratios, and degree of lipid peroxidation to (1) assess the effects of *Trichoderma asperellum* on the physical and chemical characteristics of the maize seedling rhizosphere in a saline–alkaline soil and (2) evaluate the effects of *T. asperellum* on the AsA–GSH cycle antioxidant and growth characteristics of maize seedling roots under saline–alkaline soil stress and elucidate the mechanism underlying the improvement of the rhizosphere environment and promotion of plant AsA–GSH cycle antioxidant defense system by *T. asperellum*.

## Materials and methods

### Experimental materials and design

The experiment was conducted at the Key Laboratory of Modern Agricultural Cultivation and Crop Germplasm Improvement of Heilongjiang Province, Daqing (46° 58′ N; 125° 03′ E, 150 m a.s.l.), China. Two typical and widely-grown commercial maize hybrids with differing performance on saline–alkaline soils were screened in the laboratory: ‘Jiangyu 417’ (‘JY417’) is a highly salt-tolerant cultivar, and ‘Xianyu 335’ (‘XY335’) is a salt-sensitive cultivar. Unbroken maize seeds (germination rate > 90%) of uniform size were selected and surface-sterilized with 10% NaClO solution for 10 min. Then, the seeds were rinsed with sterile distilled water, air-dried, and placed in an incubator at 25 °C in the dark for two days to promote germination. Seeds with the same sprout length were selected and transplanted into plastic pots (12 × 11 cm, width × height)^7,22^. Soil (pH 9.30) obtained from a typical in situ saline–alkaline area of Daqing was used to fill the pots after air-drying. Each plastic pot was filled with 700 g of the experimental soil and each treatment included ten replicate pots with each pot containing five seedlings. *T. asperellum* was used to treat the seedlings. First, *T. asperellum* (Genbank accession: KJ541741) was activated in PDA media 28 ± 2 °C at 185 r min^−1^ and then prepared as a spore suspension (1 × 10^9^ colony-forming units mL^−1^), which was inoculated onto a sterilized solid matrix (1:20, v/w) and incubated at 28 °C for 10 days. A concentrated *T. asperellum* spore suspension (1 × 10^9^ spores L^−1^) was prepared by adding 200 mL of the suspension to each liter of soil to obtain the following treatments: 1 × 10^3^ (T1), 1 × 10^6^ (T2), and 1 × 10^9^ (T3) spores L^−1^. A control was prepared, which consisted of 200 mL of the vehicle without spores. The strains used in this work were selected according to their biocontrol and/or plant-growth promotion activities as determined in previous experiments carried out in our laboratory^7,22^. The experiment was conducted in a semi-controlled growth chamber adjusted to 25/20 °C day/night temperature and a 16 h photoperiod under a photosynthetic photon flux density of 1000 µmol m^−2^ s^−1^. Tap water was supplied daily to maintain soil moisture. The basic physicochemical properties of the experimental soil are listed in Table S1.

### Sample collection and pretreatment

Maize seedlings were taken when the three heart-shaped leaf stage had been reached and the fourth leaf was still developing to show the persistent effect of *T. asperellum* on growth behavior (the 27th day after *T. asperellum* application). At this point, plant height, leaf dry weight, and leaf relative water content were measured. The seedlings were five plants were randomly selected and removed from the soil. Rhizosphere soil was sampled from each treatment and transferred to the laboratory, sieved through a 2 mm mesh, and air-dried for analysis of rhizosphere soil physicochemical properties and enzyme activity. We defined rhizosphere soil as that soil 0.5 cm away from any root structure (i.e., fine or coarse) that remained attached to the root zone after the plant/soil complexes were excavated, laterally shaken, and moderate pressure was applied by hand to the soil aggregates. If the soil aggregates remained attached to the roots after this procedure, they were considered rhizosphere soil^23^.

### Determination of soil characteristics

Data on soil characteristics were collected as previously described in Fu et al.^24^, by measuring pH, organic matter (OM), available nitrogen (AN), available phosphorous (AP), and available potassium (AK). Soil pH was determined using a glass combination electrode with a soil:water ratio of 1:1^25^. Soil OM was determined using the K_2_Cr_2_O_7_-H_2_SO_4_ digestion method; AN was extracted with 1 M KCl and analyzed using the cadmium reduction method, while AP was extracted with a 0.5 M NaHCO_3_ solution with the pH adjusted to 8.5, and AK was extracted with neutral 1 N NH_4_OAc^26^.

Soil hydrogen peroxidase activity was determined using the KMnO_4_ titration method^27^. The activities of sucrase, urease, and alkaline phosphatase were assayed based on the release and quantitative determination of the product glucose, NH_3_-N, and P_2_O_5_, respectively. Briefly, approximately 5 g of air-dried soil samples were incubated in 15 mL of 8% (w/v) sucrose solution^27^, 15 mL of 10% (w/v) urea solution^27^, and 20 mL of 0.5% (w/v) disodium phenyl phosphate solution^28^, as required, in a suitable buffer for 24 h at 37 °C, and spectrophotometric measurements were performed at 508, 578, or 660 nm, respectively^27^.

The soil:water ratio was adjusted to 1:5 for all crushed soil samples in preparation for the leaching solution. Each glass bottle containing the soil–water mixture was placed on an oscillator for 30 min to thoroughly dissolve the soil salts. Then, the bottle contents were allowed to settle for 24 h to obtain a clarified solution. Soil HCO_3_^−^ concentrations were determined by titrimetric hydrochloric acid methods, soil Cl^−^ concentration titration was carried out using standard AgNO_3_, and soil SO_4_^2−^ concentrations were measured using an Hucoa-Elsoss dionex 2000I/SP ionic chromatographer^29^. Soil Mg^2+^ and Ca^2+^ concentrations were measured with disodium dihydrogen ethylenediamine tetraacetate (EDTA) using a murexide indicator for calcium and an eriochrome black indicator for calcium and magnesium together^29^. Soil Na^+^ and K^+^ concentrations were determined in the filtrate using flame photometry^29^. SAR was calculated using the following equation:

$${\text{SAR}} = {{\left[ {{\text{Na}}^{ + } } \right]} \mathord{\left/ {\vphantom {{\left[ {{\text{Na}}^{ + } } \right]} {\sqrt[]{{([Ca^{2 + } ] + [Mg^{2 + } ])/2}}}}} \right. \kern-\nulldelimiterspace} {\sqrt[]{{([Ca^{2 + } ] + [Mg^{2 + } ])/2}}}},$$

where SAR was the sodium adsorption ratio (cmol kg^−1^)^0.5^, and [Na^+^], [Ca^2+^], and [Mg^2+^] were the respective concentrations in solution^18^.

### Analysis of maize roots

To separate the soil from the roots, the XY335 and JY417 maize seedling root samples described in the *Sample collection and pretreatment* section were soaked and rinsed with water over three layers of gauze cloth laid out at the bottom of a sink, and fine roots were then collected from the gauze cloth. A portion of each root sample was dried and frozen in liquid nitrogen. Roots were stored at − 80 °C until the antioxidant enzyme activity assays^7^. Frozen root tissue (1 g) was homogenized with 10 mL 0.1 M potassium phosphate buffer (pH 7.0), containing 0.1 mM EDTA-Na_2_, 0.5 mM ascorbate, and 1% polyvinyl polyvinylpyrrolidone in an ice bath^7^. The homogenate was filtered and centrifuged at 28,710 × *g* and 4 °C for 10 min, and the supernatant was used for the protein content and antioxidant enzyme activity determination^7^.

The activities of monodehydroascorbate reductase (MDHAR) and dehydroascorbate reductase (DHAR) were determined as described by Li et al.^30^. Meanwhile, on the 27th day after *T. asperellum* application the hydrogen peroxide (H_2_O_2_), malondialdehyde (MDA), superoxide anions (O_2_^−^), soluble sugars, soluble protein, proline, ascorbate peroxidase (APX), glutathione reductase (GR), AsA, and GSH contents in XY335 and JY417 roots were determined as described by Fu et al.^7^. Antioxidant enzyme activities were indicated as U mg^−1^ (enzyme activity unit number per mg protein).

The remaining root samples were used for the analysis of root activity, root volume, weight, and root superficial area as previously described^24^. Root activity was determined in a portion of the root sample using the 2,3,5-triphenyl tetrazolium chloride method^31^. The total volume of the root system was determined using the drainage method, and the superficial area of the root system was determined using the methylene blue adsorption test^24^. To determine the dry weight, root samples were weighed to determine the fresh weight, dried in a forced air oven at 105 °C for 30 min, and then dried to a constant weight at 75 °C. All of the above indicators were assessed five times.

### Statistical analysis

In this study, data were presented as means (n = 5) ± standard deviation (SD). To detect differences among treatments, the least significant difference (LSD) test was used at a significance level of P = 0.05, with C and T indicating cultivars and treatments of the tables, respectively. LSD tests and Pearson's correlations were calculated in SPSS 21.0 software package (Chicago, IL).

### Study statement

This study was conducted in compliance with relevant institutional, national, and international guidelines and legislation.

## Results

### Effects of *T. asperellum* on salt ion content, sodium adsorption ration, and pH of maize seedlings under saline–alkaline stress

After applying spore suspensions of *T. asperellum* at different concentrations, we observed significant increases in the soil contents of Ca^2+^, Mg^2+^, and K^+^ relative to those in the control, whereas, Na^+^, HCO_3_^−^, Cl^−^, and SO_4_^2−^ contents significantly decreased (Table 1). Thus, increasing *T. asperellum* spore densities in suspension effectively regulated the soil ion balance in the rhizosphere of maize seedlings, and all ions showed significant differences under treatment T3. Compared with those in the control, T3 significantly reduced the Na^+^ and HCO_3_^−^ contents by 19.46% and 35.87% in XY335, and 20.02% and 36.29% in JY417, respectively, with an effect more pronounced than that with treatments T1 and T2. Although the Cl^−^ and SO_4_^2−^ contents were low, their variation patterns were similar to that of HCO_3_^−^ content. Overall, however, the composition of ions in the rhizosphere of maize seedlings was improved by the *T. asperellum* treatment.

**Table 1:** Influence of *T. asperellum* on salt ion content, sodium adsorption ration (SAR), and pH value of maize seedlings rhizosphere soil (± SD).

Cultivars	Treatment	Cation content (g kg^−1^)				Anion content (g kg^−1^)			SAR (cmol kg^−1^)	pH value
		Ca^2+^	Mg^2+^	Na^+^	K^+^	HCO_3_^−^	Cl^−^	SO_4_^2−^		
XY335	Con	0.02 ± 0.01c	0.01 ± 0.00c	0.90 ± 0.01a	0.01 ± 0.00c	0.13 ± 0.00a	0.07 ± 0.00a	0.11 ± 0.00a	7.51 ± 0.94a	9.26 ± 0.05a
	T1	0.03 ± 0.00b	0.02 ± 0.01b	0.86 ± 0.01b	0.02 ± 0.01b	0.12 ± 0.00b	0.06 ± 0.00b	0.09 ± 0.01b	5.44 ± 0.45b	8.97 ± 0.13b
	T2	0.04 ± 0.00b	0.02 ± 0.00b	0.83 ± 0.03b	0.02 ± 0.00b	0.10 ± 0.00c	0.06 ± 0.00c	0.09 ± 0.01b	4.61 ± 0.33b	8.88 ± 0.04b
	T3	0.06 ± 0.01a	0.03 ± 0.00a	0.73 ± 0.02c	0.03 ± 0.00a	0.08 ± 0.00d	0.05 ± 0.00d	0.07 ± 0.00c	3.45 ± 0.14c	8.73 ± 0.06c
JY417	Con	0.03 ± 0.01d	0.01 ± 0.00c	0.88 ± 0.01a	0.01 ± 0.01d	0.12 ± 0.00a	0.07 ± 0.00a	0.11 ± 0.00a	6.42 ± 0.38a	9.15 ± 0.07a
	T1	0.04 ± 0.01c	0.02 ± 0.00b	0.84 ± 0.02b	0.02 ± 0.00c	0.11 ± 0.00b	0.06 ± 0.00b	0.09 ± 0.01b	4.96 ± 0.42b	8.96 ± 0.08b
	T2	0.05 ± 0.00b	0.02 ± 0.00b	0.80 ± 0.03c	0.03 ± 0.00b	0.09 ± 0.00c	0.05 ± 0.00c	0.08 ± 0.01b	4.08 ± 0.28c	8.82 ± 0.04c
	T3	0.07 ± 0.00a	0.04 ± 0.00a	0.72 ± 0.01d	0.04 ± 0.01a	0.08 ± 0.00d	0.04 ± 0.00d	0.06 ± 0.01c	3.15 ± 0.03d	8.66 ± 0.04d
	ANOVA									
	C	*	NS	*	*	*	**	*	**	*
	T	**	**	**	**	**	**	**	**	**
	C × T	NS	NS	NS	NS	*	NS	NS	NS	NS

Con, T1, T2, and T3 indicate 0, 1 × 10^3^, 1 × 10^6^, and 1 × 10^9^ spores L^−1^ suspension, respectively. Soil salt ion content, SAR, and pH were measured on the 27th day after *T. asperellum* application. C and T indicated cultivars and treatments, respectively. Different letters within a column indicated significant differences at 5% probability level, and the numerical value represented the mean value of five repeats. Differences between treatments were calculated for each particular cultivar. NS, not significant. * and **, significant at the 0.05 and 0.01 probability level, respectively.

As shown in Table 1, compared with those in the control, *T. asperellum* treatment significantly reduced the soil pH and SAR values, although with no significant cultivar × treatment interaction effects (P < 0.05). Under saline–alkaline stress, pH was 9.26 and 9.15 in soils planted with XY335 and JY417, respectively, and we found that for both maize cultivars, the pH and SAR values in the rhizosphere soil decreased with an increasing concentration of *T. asperellum* spores. Under treatment T3, pH was 8.73 and 8.66, while SAR was 3.45 and 3.15 for XY335 and JY417, respectively, which were all significantly different from those recorded under treatments T1 and T2. However, although soil pH and SAR for XY335 did not differ significantly between treatments T1 and T2, we detected significantly different responses between these two treatments in JY417. Further, pH and SAR values in JY417 were lower than those in XY335, indicating that JY417 showed a certain degree of tolerance to saline–alkaline stress.

### Effects of *T. asperellum* on the nutrient contents of maize seedlings in rhizosphere soil

As shown in Table 2, compared with those in the control, *T. asperellum* treatment significantly increased soil nutrient parameters, although without any significant cultivar × treatment interaction effects (P < 0.05), and with the rhizosphere soil nutrient content for JY417 being higher than that for XY335. Similarly, soil chemical parameters in the rhizosphere soil were significantly improved, being higher after treatment with *T. asperellum* than those in the control. Furthermore, nutrient contents increased with increasing *T. asperellum* spore concentration, showing notable differences between treatments T1 and T2. Compared with those in the control, there were significant increases in the soil contents of OM, AN, AP, and AK: 65.32%, 23.80%, 123.60%, and 46.09% in XY335, and 67.42%, 21.14%, 109.94%, and 48.50% in JY417, respectively, in response to treatment T3.

**Table 2:** Influence of *T. asperellum* on the nutrient contents of maize seedling rhizosphere soil (± SD).

Cultivars	Treatment	Organic matter (g kg^−1^)	± Con%	Available N (mg kg^−1^)	± Con%	Available P (mg kg^−1^)	± Con%	Available K (mg kg^−1^)	± Con%
XY335	Con	15.66 ± 1.40c	–	101.04 ± 2.99c	–	7.84 ± 2.00d	–	81.18 ± 8.47c	–
	T1	19.88 ± 3.85b	26.95	113.07 ± 4.84b	11.91	10.97 ± 0.51c	39.92	95.88 ± 8.34b	18.11
	T2	21.55 ± 1.46b	37.61	114.24 ± 6.30b	13.06	12.34 ± 0.74b	57.40	98.95 ± 4.75b	21.89
	T3	25.89 ± 1.00a	65.32	125.09 ± 6.25a	23.80	17.53 ± 0.91a	123.60	118.60 ± 10.93a	46.10
JY417	Con	16.76 ± 2.38c	–	104.90 ± 1.84d	–	8.95 ± 0.76d	–	83.03 ± 7.25c	–
	T1	21.99 ± 2.50b	31.21	115.83 ± 2.68c	10.42	11.74 ± 0.36c	31.17	101.62 ± 7.12b	22.39
	T2	24.56 ± 1.04b	46.54	118.61 ± 1.90b	13.07	13.91 ± 0.69b	55.42	105.49 ± 7.67b	27.05
	T3	28.06 ± 1.28a	67.42	127.08 ± 2.35a	21.14	18.79 ± 1.29a	109.94	123.30 ± 13.40a	48.50
	ANOVA								
	C	*		*		**		NS	
	T	**		**		**		**	
	C × T	NS		NS		NS		NS	

Con, T1, T2, and T3 indicate 0, 1 × 10^3^, 1 × 10^6^, 1 × 10^9^ spores L^−1^ suspension, respectively. Soil nutrient contents were measured on the 27th day after *T. asperellum* application. C and T indicated cultivars and treatments, respectively. Different letters within a column represented significant difference at 5% probability level, and the numerical value indicated the mean value of five repeats. Differences between treatments were calculated for each particular cultivar. NS, not significant. * and **, significant at the 0.05 and 0.01 probability level, respectively.

### Effects of *T. asperellum* on the enzyme activity of maize seedlings in rhizosphere soil

As shown in Table 3, there was a significant increase in soil enzyme activities in XY335 and JY417 rhizosphere soil after *T. asperellum* application compared to those in the control, although no significant cultivar × treatment interaction effects were detected, except for alkaline phosphatase activity (P < 0.05). Soil enzyme activities in JY417 rhizosphere soil tended to be generally higher than those in XY335 soil. Moreover, we noted that soil enzyme activities were differently enhanced by an increase in *T. asperellum* concentration from T1 and T2. However, the highest soil enzyme activities were recorded under treatment T3 in each case, which were significantly higher than those in the other treatments. Compared with those in the control, the T3 treatment significantly increased soil urease, phosphatase, sucrase, and hydrogen peroxidase activities by 38.24%, 43.48%, 38.13%, and 55.40% in XY335, and 37.83%, 51.89%, 29.43%, and 52.79% in JY417, respectively. Thus, *T. asperellum* treatment can significantly increase soil enzyme activities and improve the soil physical and chemical environment in the rhizosphere of maize seedlings.

**Table 3:** Influence of *T. asperellum* on the enzyme activity of maize seedlings rhizosphere soil (± SD).

Cultivars	Treatment	Urease (NH_3_^−^N mg g^−1^)	± Con%	Alkaline phosphatase (Phenol mg g^−1^)	± Con%	Sucrose (Glu mg g^−1^)	± Con%	Hydrogen peroxidase (0.1 N KMnO_4_ ml g^−1^)	± Con%
XY335	Con	0.35 ± 0.01c	–	11.89 ± 0.56d	–	25.20 ± 1.87c	–	2.13 ± 0.02c	–
	T1	0.40 ± 0.00b	17.65	13.94 ± 0.11c	17.24	30.16 ± 0.61b	19.68	2.50 ± 0.12b	17.37
	T2	0.42 ± 0.02b	23.53	15.91 ± 0.38b	33.81	31.60 ± 0.24b	25.40	3.00 ± 0.04b	40.85
	T3	0.47 ± 0.02a	38.24	17.06 ± 0.14a	43.48	34.81 ± 0.94a	38.13	3.31 ± 0.13a	55.40
JY417	Con	0.37 ± 0.01c	–	12.64 ± 0.18d	–	28.47 ± 1.64c	–	2.33 ± 0.09d	–
	T1	0.41 ± 0.01b	10.81	14.98 ± 0.33c	18.51	32.02 ± 0.04b	12.47	2.64 ± 0.09c	13.30
	T2	0.44 ± 0.02b	18.92	16.51 ± 0.17b	30.62	33.95 ± 1.25b	19.25	3.22 ± 0.11b	38.20
	T3	0.51 ± 0.02a	37.83	19.20 ± 0.36a	51.89	36.85 ± 1.15a	29.43	3.56 ± 0.07a	52.79
	ANOVA								
	C	**		**		**		**	
	T	**		**		**		**	
	C × T	NS		**		NS		NS	

Con, T1, T2, and T3 indicate 0, 1 × 10^3^, 1 × 10^6^, 1 × 10^9^ spores L^−1^ suspension, respectively. Soil enzyme activity was measured on the 27th day after *T. asperellum* application. C and T indicated cultivars and treatments, respectively. Different letters within a column represented significant differences at 5% probability level, and the numerical value was the mean value of five repeats. Differences between treatments were calculated for each particular cultivar. NS, not significant. * and **, significant at the 0.05 and 0.01 probability level, respectively.

### Growth promotion in maize seedlings treated with *T. asperellum* under saline–alkaline soil stress

Compared to those in the control, *T. asperellum* treatment significantly increased the maize seedling growth under saline–alkaline stress by significantly enhancing all measured seedling variables, although we detected no significant cultivar × treatment interaction effects (Table 4, Table S2). Moreover, the dry weight of the root system and root relative water content, volume, superficial area, and activity in seedlings treated with *T. asperellum* were significantly higher than those in the control, and the effect was concentration dependent.

**Table 4:** Influence of *T. asperellum* on the root growth characteristic of maize seedlings rhizosphere soil (± SD).

Cultivars	Treatment	Root dry weight (g plant^−1^)	± Con%	Root relative water content (%)	± Con%	Root volume (cm^3^ plant^−1^)	± Con%	Root surface (m^2^ plant^−1^)	± Con%	Root activity (mg g^−1^ h^−1^)	± Con%
XY335	Con	0.05 ± 0.003c	–	81.89 ± 1.12d	–	17.37 ± 0.31d	–	9.58 ± 0.17d	–	10.72 ± 0.52c	–
	T1	0.06 ± 0.001b	18.00	84.78 ± 0.10c	3.53	27.29 ± 0.60c	57.11	15.01 ± 0.33c	56.68	14.30 ± 1.09b	33.40
	T2	0.07 ± 0.003b	32.00	86.88 ± 0.27b	6.09	30.95 ± 0.55b	78.18	17.02 ± 0.30b	77.66	15.50 ± 1.14b	44.59
	T3	0.08 ± 0.003a	58.01	89.25 ± 0.53a	8.99	34.47 ± 0.75a	98.45	18.96 ± 0.41a	97.91	20.06 ± 1.95a	87.13
JY417	Con	0.06 ± 0.005c	–	83.13 ± 1.11d	–	20.06 ± 1.56d	–	11.03 ± 0.86d	–	13.52 ± 0.36d	–
	T1	0.07 ± 0.004b	14.04	85.54 ± 0.99c	2.90	29.58 ± 0.84c	47.46	16.27 ± 0.46c	47.51	16.69 ± 0.91c	23.45
	T2	0.08 ± 0.003b	31.58	88.10 ± 0.40b	5.98	34.38 ± 1.08b	71.39	18.91 ± 0.59b	71.44	19.50 ± 1.26b	44.23
	T3	0.08 ± 0.002a	42.11	90.54 ± 0.13a	8.91	39.56 ± 0.95a	97.21	21.76 ± 0.52a	97.28	22.42 ± 2.33a	65.83
	ANOVA										
	C	**		**		**		**		**	
	T	**		**		**		**		**	
	C × T	NS		NS		*		*		NS	

Con, T1, T2, and T3 indicate 0, 1 × 10^3^, 1 × 10^6^, 1 × 10^9^ spores L^−1^ suspension, respectively. Root growth characteristics were measured on the 27th day after *T. asperellum* application. C and T indicated cultivars and treatments, respectively. Different small letter within a column represented significant differences at 5% probability level, and the numerical value was the mean of five repeats. Differences between treatments were calculated for each particular cultivar. NS, not significant. * and **, significant at the 0.05 and 0.01 probability level, respectively.

The recorded values for all variables increased with an increasing concentration of *T. asperellum*, with the growth-enhancing effects of the fungus on XY335 being more apparent than those on JY417 seedlings.

### Effects of *T. asperellum* on non-enzymatic system contents in the roots of maize seedlings cultured in a saline–alkaline soil

Compared with those control, the soluble sugar and proline contents significantly increased in the roots of *T. asperellum*-treated maize seedlings (P < 0.05), particularly in those receiving the T3 treatment, which promoted the largest accumulation of these substances. These increases in osmoregulatory substances may explain how treatment with *T. asperellum* induces systemic resistance to saline–alkaline stress in maize seedlings (Table 5).

**Table 5:** Influence of *T. asperellum* on the content of non-enzymatic systems in the roots of maize seedlings in saline–alkaline soil (± SD).

Cultivars	Treatment	Proline content (ug g^−1^ FW)	± Con%	Soluble sugar content (mg g^−1^ FW)	± Con%	GSH/GSSG content	± Con%	ASA/DHA content	± Con%
XY335	Con	70.13 ± 1.42d	–	55.38 ± 1.17c	–	3.83 ± 0.25d	–	3.54 ± 0.05d	–
	T1	82.10 ± 4.02c	17.07	68.72 ± 2.15b	24.09	5.51 ± 0.15c	43.86	5.01 ± 0.25c	41.53
	T2	101.93 ± 1.03b	45.34	71.14 ± 2.17b	28.46	6.47 ± 0.09b	68.93	6.46 ± 0.32b	82.49
	T3	115.68 ± 3.15a	64.95	85.15 ± 1.36a	53.76	7.21 ± 0.10^a^	88.25	11.41 ± 0.12^a^	222.32
JY417	Con	77.52 ± 2.35d	–	62.15 ± 2.30d	–	4.02 ± 0.13d	–	4.83 ± 0.22d	–
	T1	94.82 ± 3.10c	22.32	74.15 ± 1.31c	19.31	5.79 ± 0.21c	44.03	6.05 ± 0.28c	25.26
	T2	111.54 ± 1.21b	43.89	79.04 ± 1.33b	27.18	6.83 ± 0.11b	69.90	8.19 ± 0.56b	69.57
	T3	126.41 ± 2.13a	63.07	88.77 ± 1.52a	42.83	7.53 ± 0.27^a^	87.31	13.24 ± 0.73^a^	174.12
	ANOVA								
	C	**		**		**		**	
	T	**		**		**		**	
	C × T	NS		NS		NS		**	

Con, T1, T2, and T3 indicate 0, 1 × 10^3^, 1 × 10^6^, 1 × 10^9^ spores L^−1^ suspension, respectively. Non-enzymatic system contents were measured on the 27^th^ day after *T. asperellum* application. C and T indicated cultivars and treatments, respectively. Different letters within a column represented significantly different at 5% probability level, and the numerical value was the mean value of five repeats. Differences between treatments were calculated for each particular cultivar. NS, not significant. * and **, significant at the 0.05 and 0.01 probability level, respectively.

To elucidate the effect of the AsA–GSH cycle on alleviating oxidative stress in maize seedling roots under saline–alkaline stress, we examined the GSH/GSSG and AsA/DHA ratios (Table 5), showing that these two ratios were significantly reduced under saline–alkaline stress, but significantly increased in response to treatment with increasing concentrations of *T. asperellum* spores. We also detected significant cultivar × treatment interaction effects on AsA/DHA. Thus, the AsA–GSH cycle appeared to play an important role in controlling the saline–alkaline tolerance induced by *T. asperellum*, with XY335 performing better than JY417 in this regard.

### Effects of *T. asperellum* on antioxidant enzyme activities in the roots of maize seedlings cultured in saline–alkaline soil

To determine whether the antioxidant enzyme system plays a role in the *T. asperellum*-induced saline–alkaline tolerance of maize seedlings, we determined the activities of enzymes involved in the AsA–GSH cycle (Table 6). APX, MDHAR, DHAR, and GR significantly increased in the XY335 and JY417 maize seedling roots with increasing concentration of *T. asperellum* under saline–alkaline stress, with a significant cultivar × treatment interaction effect on APX. Under treatment T3, APX, MDHAR, DHAR, and GR activities increased by 75.02%, 37.66%, 40.26%, and 76.09% in XY335, and 67.18%, 34.41%, 38.95%, and 58.38% (P < 0.05) in JY417, respectively, compared with those of the control. Thus, overall, *T. asperellum* can promote the seedlings of both cultivars by enhancing antioxidation enzyme activities, which is beneficial in terms of coping with the generation of excess ROS associated with saline–alkaline stress.

**Table 6:** Influence of *T. asperellum* on the antioxidant enzyme activities in the roots of maize seedlings in saline–alkaline soil (± SD).

Cultivars	Treatment	APX activity (U min^−1^ mg^−1^ proteing)	± Con%	MDHAR activity (U min^−1^ mg^−1^ proteing)	± Con%	DHAR activity (U min^−1^ mg^−1^ proteing)	± Con%	GR activity (U min^−1^ mg^−1^ proteing)	± Con%
XY335	Con	25.30 ± 2.34d	–	43.30 ± 1.83d	–	52.85 ± 3.56d	–	3.68 ± 1.40d	–
	T1	33.25 ± 5.37c	31.42	49.52 ± 1.43c	14.36	62.29 ± 2.57c	17.86	4.35 ± 1.25c	18.21
	T2	38.75 ± 2.46b	53.16	53.75 ± 1.06b	24.13	68.72 ± 2.15b	30.03	5.30 ± 2.01b	44.02
	T3	44.28 ± 2.44a	75.02	59.61 ± 1.39a	37.66	74.13 ± 1.78a	40.26	6.48 ± 1.31a	76.09
JY417	Con	30.47 ± 3.71d	–	47.20 ± 1.47d	–	56.66 ± 1.87d	–	4.95 ± 0.93d	–
	T1	35.67 ± 2.67c	17.07	51.67 ± 1.28c	9.47	64.42 ± 1.62c	13.70	5.34 ± 1.01c	7.88
	T2	42.73 ± 3.70b	40.24	57.39 ± 2.29b	21.59	71.36 ± 1.85b	25.94	6.43 ± 0.67b	29.90
	T3	50.94 ± 5.93a	67.18	63.44 ± 1.92a	34.41	78.73 ± 1.64a	38.95	7.84 ± 0.35a	58.38
	ANOVA								
	C	**		**		**		**	
	T	**		**		**		**	
	C × T	**		NS		NS		NS	

Con, T1, T2, and T3 indicate 0, 1 × 10^3^, 1 × 10^6^, 1 × 10^9^ spores L^−1^ suspension, respectively. Antioxidant enzyme activities were measured on the 27th day after *T. asperellum* application. C and T indicated cultivars and treatments, respectively. Different letters within a column represented significant differences at 5% probability level, and the numerical value was the mean value of five repeats. Differences between treatments were calculated for each particular cultivar. NS, not significant. * and **, significant at the 0.05 and 0.01 probability level, respectively.

### Effects of *T. asperellum* on the reactive oxygen species accumulation and oxidation parameters in the roots of maize seedlings grown in saline–alkaline soils

When analyzing the effect of *T. asperellum* on ROS clearance, we found that the contents of H_2_O_2_, O_2_^−^, and MDA in the roots of maize seedlings growing under control conditions were significantly higher than those in the other treatments, with significant cultivar × treatment interaction effects (P < 0.05; Table S3). After 27 days of treatment, H_2_O_2_, O_2_^−^, and MDA contents in the roots of maize seedlings had decreased significantly in response to treatment with increasing concentrations of *T. asperellum*, indicating that *T. asperellum* can enhance ROS clearance in the roots of maize seedlings by affecting both enzymatic and non-enzymatic systems (i.e., antioxidant enzyme activity and osmoregulation, respectively).

### Relationships between the AsA–GSH cycle enzyme activity and soil characteristics

Pearson’s correlation analysis was used to evaluate the relationships between the AsA–GSH cycle enzyme activity and soil characteristics (Table 7). The AsA–GSH cycle enzyme activity was significantly correlated with soil characteristics, exhibiting a positive correlation with soil nutrient and a negative correlation with soil pH and SAR.

**Table 7:** Pearson's correlations between soil characteristics and the AsA–GSH cycle enzyme activity.

	GR	APX	MDHAR	DHAR
Urease	0.905**	0.956**	0.924**	0.945**
Sucrose	0.909**	0.953**	0.903**	0.936**
Hydrogen peroxidase	0.925**	0.978**	0.963**	0.942**
Alkaline phosphatase	0.924**	0.985**	0.970**	0.962**
Available N		0.891**	0.904**	0.920**
Available P	0.910**	0.947**	0.923**	0.913**
Available K	0.821*		0.853*	0.837*
Organic matter	0.869**	0.909**	0.876**	0.880**
pH	− 0.856*	− 0.937**	− 0.936**	− 0.951**
SAR	− 0.855*	− 0.937**	− 0.934**	− 0.933**

**P < 0.01, *P < 0.05.

## Discussion

In our study sites, we showed that soluble salt contents in the soil contributed to high soil pH and SAR values. However, compared to those of the control, Na^+^, HCO_3_^−^, Cl^−^, and SO_4_^2−^ contents in soil were significantly reduced through treatment with increasing concentrations of *T. asperellum* spore suspensions. Concomitantly, the contents of Ca^2+^, Mg^2+^, and K^+^ in the rhizosphere soil of treated seedlings were significantly higher than those in the control. Moreover, *T. asperellum* treatment promoted significant reductions in soil pH and SAR values and alleviated Na^+^ toxicity to maize seedlings, particularly at the higher spore concentrations examined. These results were in line with the reports by Vinale et al.^32^, who found that, as part of their normal metabolism, several *Trichoderma* strains produce organic acids with a certain buffering effect on soil pH. These effects could be attributed to a promotion of the soil microbiota metabolic processes by *T. asperellum*, whereby OM is converted to humus, which can adsorb excess salt ions in the soil to a certain extent^33,34^. Furthermore, *T. asperellum* improved the chemical structure of the saline–alkaline soil, thereby increasing its permeability and promoting water–salt balance in the saline–alkaline soil, subsequently reducing salt accumulation and lowering soil pH. Notably, soluble salt contents in the soil differed between the two examined maize cultivars, with XY335 salt contents performing better than those in JY417, indicating that different maize cultivars differed in their capacity to absorb salt during growth for use in biosynthesis.

*Trichoderma* can increase soil OM and improve plant absorption of soil available nutrients^35^, with soil enzymes playing an important role in the transformation and circulation of soil nutrients^36^. Our results revealed significant reductions in soil OM and available nutrient contents in the rhizosphere soil of the two maize cultivars under control condition, whereas the contents of these soil constituents increased upon treating seedlings with suspensions of *T. asperellum* spores. Moreover, we noted that the soil OM and available nutrient contents increased gradually with increasing concentrations of *T. asperellum*. In addition, the increase in AP was higher than that of alkali-hydrolyzed nitrogen and AK under *T. asperellum* treatment, these observations indicated that *Trichoderma* can convert unavailable soil inorganic phosphorus to AP, which was consistent with the findings of previous studies^37^. Furthermore, we observed an increase in soil nitrogen in response to *Trichoderma* treatment, which may be related to an increase in microbial nitrogen fixation promoted by *Trichoderma*^38^. Additionally, saline–alkaline stress significantly inhibited soil enzyme activity, and this effect was significantly alleviated in response to the application of increasing *T. asperellum* spore concentrations. Notably, the soil nutrient contents and enzyme activities in the rhizosphere of maize cultivar JY417 were higher than those in XY335, thereby highlighting the cultivar dependence of *T. asperellum* effects on the physicochemical properties of rhizosphere soils. Therefore, *T. asperellum* application effectively promoted soil enzyme activities in the rhizosphere, increased soil nutrient content, and improved the rhizosphere soil chemical properties, thereby alleviating saline–alkaline soil stress on maize seedlings.

Fungi in the genus *Trichoderma* have been reported to promote the growth of *Triticum aestivum* L.^39^, *Brassica juncea* L.^40^, *Zea mays* L., and *Oryza sativa* L.^19^. Moreover, the application of *T. asperellum* spore suspensions increased the dry weight of maize seedlings in this study (Table S2). In addition to improving plant growth (e.g., plant height), *T. asperellum* treatment can promote root development (Table 4). Given that the root tips and surface are the sites of nutrient uptake in maize, *T. asperellum* could be promoting maize seedling growth via stimulation of the root system growth, leading to an increase in the nutrient uptake capacity. Root growth and vigor directly affect plant growth, nutrition, and crop yield, and root activity serves as one of the main indicators of root function^41^. Therefore, we analyzed the root activity of maize seedlings treated with *T. asperellum* and found a significant rhizosphere interaction between *T. asperellum* and maize seedlings. Moreover, treatment with *T. asperellum* enhanced root features in both maize cultivars examined (Table 4).

We also demonstrated that *T. asperellum* application can enhance maize seedling resistance to saline–alkaline stress by inducing non-enzymatic changes in soluble sugars and proline in the roots (Table 5). Plants accumulate osmoregulatory substances and promote osmotic balance under saline–alkaline stress, which leads to enhanced tolerance to dehydration^21,42^. Thus, *T. asperellum* improves osmotic regulation by decreasing osmotic pressure and maintaining the water absorption capacity of the cells, thereby reducing the adverse effects of saline–alkaline stress by balancing osmotic potential, ultimately enhancing the saline–alkaline tolerance of maize seedlings.

*Trichoderma* treatment also induced antioxidant enzyme activities, and thereby minimized the oxidative damage caused by ROS under conditions of saline–alkaline stress. Enhanced antioxidation enzyme activity can protect plant tissues from oxidative damage to membranes under saline–alkaline stress, thereby reducing saline–alkaline toxicity and enhancing plant growth. In this study, different *T. asperellum* concentrations increased the activities of APX, MDHAR, DHAR, and GR in the roots of maize seedlings (Table 6). We suspect that enhanced MDHAR and DHAR activities may have contributed to an increase in AsA content and a decrease in DHA content under saline–alkaline stress. Under normal conditions, ROS, including H_2_O_2_, O_2_^−^, and MDA, act as signal molecules at low concentrations. However, excessive ROS accumulation under saline–alkaline stress is detrimental to plant tissues^43^. Consistent with the changes observed in enzyme activities, *T. asperellum* treatment promoted significant reductions in H_2_O_2_, O_2_^−^, and MDA contents under saline–alkaline stress conditions. Our data indicated that *T. asperellum* treatment enhanced the saline–alkaline tolerance of maize seedlings by inducing the root antioxidant enzyme system to remove excess ROS.

The AsA–GSH cycle is one of the major pathways through which excess H_2_O_2_ is removed from chloroplasts to neutralize the toxic effects of oxidative stress generated by this ROS^44^. AsA and GSH are two important antioxidants in plants subjected to environmental stress, whereas APX, MDHAR, DHAR, and GR are important ROS scavenging enzymes^45^. Collectively, AsA, GSH, APX, MDHAR, DHAR, and GR constitute the AsA–GSH cycle responsible for H_2_O_2_ detoxification, with stable GSH/GSSG and AsA/DHA ratios being crucial for maintaining cellular redox homeostasis under environmental stress^46^. In this study, *T. asperellum* treatment increased GSH/GSSG and AsA/DHA ratios (Table 5) in the root system of maize seedlings. Relative to those in the control, the observed ratio differences were significant at different *T. asperellum* concentrations in each of the maize cultivars examined, with XY335 performing better than JY417, which may explain *Trichoderma* promoting saline–alkaline tolerance in maize seedlings, although probably the underlying mechanisms differ for each cultivar. Nonetheless, *T. asperellum* treatment significantly alleviated all measured indices of oxidative damage caused by saline–alkaline stress in maize seedlings.

In this study, maize growth in *Trichoderma*-treated soils was better than that in the control soil and was significantly correlated with soil characteristics and AsA–GSH cycle, whose combined effect enhanced saline–alkaline stress tolerance in maize plants. These observations provide further evidence for associations of maize growth with soil characteristics and AsA–GSH cycle, which will be useful for improving crop yields in challenging saline–alkaline soils.

## Conclusions

This study investigated the role of exogenous *T. asperellum* in improving the components of the antioxidant defence system and remediation the saline–alkaline soil to enhance saline–alkaline stress tolerance in maize plants. First, the application of *T. asperellum* to saline-alkali soil reduced soil pH and SAR values, improved the nutrient content and enzyme activity of soil, which could directly promote the growth of maize. Second, *T. asperellum* activated more efficient antioxidant systems with enhanced antioxidant enzymes and improved the cellular redox status, which may be a useful strategy for lessening the accumulation of H_2_O_2_ and O_2_^−^, ensuring the structural and functional integrity of the cell membrane, and alleviating the inhibition of plant growth. The associated changes in AsA–GSH cycle enzyme activity were attributed to the general improvement in the saline–alkaline soil environment, which suggested that the effect of different doses of *Trichoderma* on maize growth might be cooperatively driven by changes in the AsA–GSH cycle enzyme and soil characteristics. Hence, *T. asperellum* can efficiently act as plant grow promoting fungi (PGPF) are in maize for successful avoidance of saline–alkaline stress damage. Accordingly, we believe that this fungus can be developed as an effective bio-fertilizer and be used to promote sustainable agricultural practices. However, there are some issues that need to be addressed in future studies, such as the efficacy of the strain of plant-growth-promoting fungi *T. asperellum* interactions with other plant species and other abiotic stresses.

## Supplementary Information

Supplementary Table S1.

Supplementary Table S2.

Supplementary Table S3.

Supplementary Information.
