# Supplementary material for: Saline–alkaline stress in growing maize seedlings is alleviated by Trichoderma asperellum through regulation of the soil environment
Source: Sci Rep. 2021 May 27;11:11152. doi: 10.1038/s41598-021-90675-9 (PMC8159927; doi:10.1038/s41598-021-90675-9)
Supplement: Supplementary file 1 — Supplementary Table S1. [file 41598_2021_90675_MOESM1_ESM.doc]

Table S1 Basic physicochemical properties of the experimental soil.

| Treatment | pH Value | Total nitrogen（g·kg^-1^） | Total phosphorus（g·kg^-1^） | Alkali-hydrolyzale nitrogen  （mg·kg^-1^） | Available phosphorus  （mg·kg^-1^） | Available potassium  （mg·kg^-1^） | Organic matter（g·kg^-1^） |
| --- | --- | --- | --- | --- | --- | --- | --- |
| Saline-alkaline Soil | 9.17 | 1.26 | 0.41 | 113.24 | 15.38 | 80.40 | 14.63 |
