# Supplementary material for: Saline–alkaline stress in growing maize seedlings is alleviated by Trichoderma asperellum through regulation of the soil environment
Source: Sci Rep. 2021 May 27;11:11152. doi: 10.1038/s41598-021-90675-9 (PMC8159927; doi:10.1038/s41598-021-90675-9)
Supplement: Supplementary file 2 — Supplementary Table S2. [file 41598_2021_90675_MOESM2_ESM.doc]

Table S2 Influence of *T. asperellum* on the growth parameters of maize seedlings under saline–alkaline stress (± SD)

| Cultivars | Treatment | Plant height（cm·plant^-1^） | ±Con% | Leaf dry weight（g·plant^-1^） | ±Con% | Leaf relative water content （%） | ±Con% |
| --- | --- | --- | --- | --- | --- | --- | --- |
| XY335 | Con | 19.67±1.52d | - | 0.07±0.00d | - | 87.03±0.58c | - |
|  | T1 | 24.87±2.51c | 26.44% | 0.09±0.00c | 32.35% | 88.85±0.56bc | 2.09% |
|  | T2 | 28.17±1.04b | 43.21% | 0.11±0.01b | 61.76% | 90.05±0.53b | 3.47% |
|  | T3 | 30.67±0.76a | 55.92% | 0.13±0.00a | 91.18% | 91.13±0.12a | 4.71% |
| JY417 | Con | 22.83±0.57d | - | 0.08±0.00d | - | 87.94±0.32c | - |
|  | T1 | 26.93±2.06c | 17.96% | 0.10±0.00c | 25.00% | 89.29±0.33c | 1.54% |
|  | T2 | 29.66±0.28b | 29.92% | 0.13±0.01b | 62.50% | 90.20±0.89b | 2.57% |
|  | T3 | 31.83±1.04a | 39.42% | 0.15±0.00a | 87.50% | 91.64±0.13a | 4.21% |
|  | ANOVA |  |  |  |  |  |  |
|  | C | ** |  | ** |  | * |  |
|  | T | ** |  | ** |  | ** |  |
|  | C×T | NS |  | NS |  | NS |  |

Note: Con, T1, T2, and T3 indicate 0, 1 × 10^3^, 1 × 10^6^, 1 × 10^9^ spores L^-1^ suspension, respectively. Root growth characteristics were measured on the 27^th^ day after *T. asperellum* application. C and T indicated cultivars and treatments, respectively. Different small letter within a column represented significant differences at 5% probability level, and the numerical value was the mean of five repeats. Differences between treatments were calculated for each particular cultivar. NS, not significant. * and **, significant at the 0.05 and 0.01 probability level, respectively.
