# Supplementary material for: Saline–alkaline stress in growing maize seedlings is alleviated by Trichoderma asperellum through regulation of the soil environment
Source: Sci Rep. 2021 May 27;11:11152. doi: 10.1038/s41598-021-90675-9 (PMC8159927; doi:10.1038/s41598-021-90675-9)
Supplement: Supplementary file 3 — Supplementary Table S3. [file 41598_2021_90675_MOESM3_ESM.doc]

Table S3 Influence of *T. asperellum* on the accumulation of ROS and on the oxidation parameters in the roots of maize seedlings in saline–alkaline soil (± SD)

| Cultivars | Treatment | H_2_O_2_ content  （umol·g^-1^ FW） | ±Con% | O_2_- content  （ug·g^-1^ FW） | ±Con% | MDA content  （umol·g^-1^ FW） | ±Con% |
| --- | --- | --- | --- | --- | --- | --- | --- |
| XY335 | Con | 138.43±4.12a | - | 24.81±2.54a | - | 6.39±0.37a | - |
|  | T1 | 101.66±5.71b | 26.56% | 16.48±2.02b | 33.58% | 4.97±0.38b | 22.22% |
|  | T2 | 71.76±3.14c | 48.16% | 13.14±2.93c | 47.04% | 3.80±0.22c | 40.53% |
|  | T3 | 54.24±2.43d | 60.82% | 9.50±1.73d | 61.71% | 3.05±0.11d | 52.27% |
| JY417 | Con | 115.51±3.51a | - | 21.73±3.45a | - | 5.23±0.23a | - |
|  | T1 | 83.81±3.57b | 27.44% | 15.54±0.57b | 28.49% | 3.97±0.62b | 24.09% |
|  | T2 | 61.96±4.19c | 46.36% | 11.90±0.29c | 45.24% | 3.52±0.29b | 32.70% |
|  | T3 | 50.06±1.66d | 56.66% | 8.37±0.82d | 61.48% | 2.87±0.71c | 45.12% |
|  | ANOVA |  |  |  |  |  |  |
|  | C | ** |  | ** |  | ** |  |
|  | T | ** |  | ** |  | ** |  |
|  | C×T | ** |  | ** |  | ** |  |

Note: Con, T1, T2, and T3 indicate 0, 1 × 10^3^, 1 × 10^6^, 1 × 10^9^ spores L^-1^ suspension, respectively. Root growth characteristics were measured on the 27^th^ day after *T. asperellum* application. C and T indicated cultivars and treatments, respectively. Different small letter within a column represented significant differences at 5% probability level, and the numerical value was the mean of five repeats. Differences between treatments were calculated for each particular cultivar. NS, not significant. * and **, significant at the 0.05 and 0.01 probability level, respectively.
